# A Tetracycline-Inducible Promoter Replacement System for *Schizosaccharomyces pombe*

**DOI:** 10.17912/micropub.biology.001759

**Published:** 2025-08-21

**Authors:** Samirul Bashir, Sasirekha Sivakumar, Nicholas Rhind

**Affiliations:** 1 Department of Biochemistry and Molecular Biotechnology, University of Massachusetts Chan Medical School, Worcester, Massachusetts, United States

## Abstract

Inducible promoters are essential tools for regulating gene expression. In fission yeast, various inducible promoter systems have been developed over the years, aiding gene function studies. A key challenge with existing promoters is their high expression in the “off ” state, with most systems showing only about a 10-fold difference between “on” and “off” conditions. A recent study introduced the *PenotetS* tetracycline promoter system, achieving nearly a 100-fold dynamic range. However, it was not designed to replace endogenous promoters. In this work, we have adapted the *PenotetS* system to enable the replacement of gene promoters directly at their endogenous genomic locations.

**Figure 1. Tetracycline promoter cassette for replacing endogenous promoters in S. pombe:**
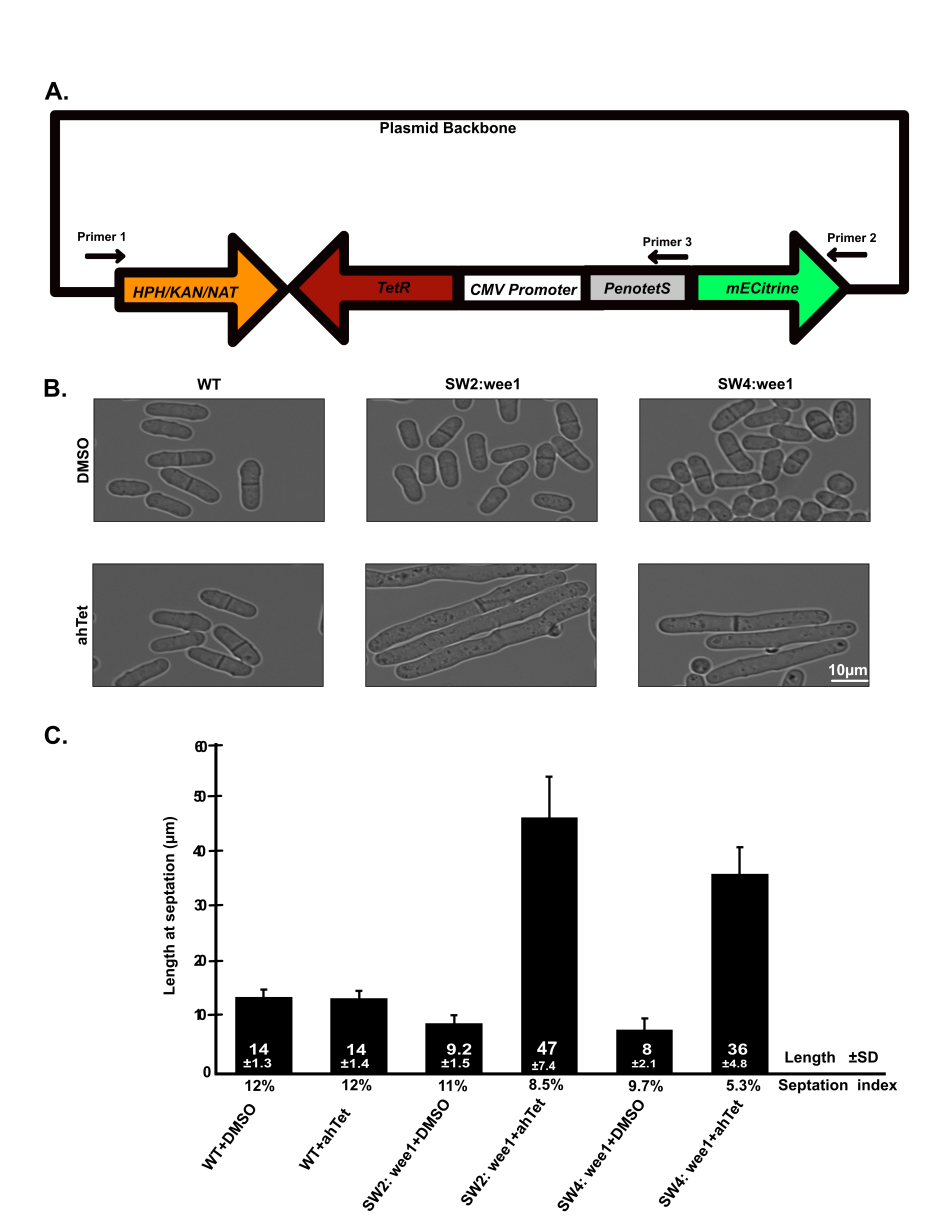
(A) A graphical representation of the tetracycline promoter construct. It includes an antibiotic-resistant cassette, either KanMX, HphMX, or NatMX, tetracycline repressor (TetR) driven by CMV promoter, a tetracycline promoter denoted by either *PenotetS* or *PenotetSW1-SW4* with the mECitrine fluorescent protein driven by the tetracycline promoter. (B) Wild-type (yFS110), *SW2::wee1* (yFS1161), and *SW4::wee1* (yFS1162) cells were grown in either DMSO only or 2.5 µg/ml ahTet overnight. (C) The length at septation and percent septation of cells shown in Figure 1B.

## Description

Controlling gene expression is essential for studying its regulation and functions. Promoters are among the most commonly used tools for this purpose. These include constitutive and inducible promoters (Russell and Hall, 1983; Russell and Nurse, 1986; Hoffman and Winston, 1989; Maundrell, 1990; Matsuyama et al., 2008; Ohira et al., 2017). A challenge with inducible promoters is their tendency for leaky expression in the “off” state, which can complicate the interpretation of specific phenotypes (Bøe et al., 2008).

In *Schizosaccharomyces pombe* , the most commonly used promoters are the *nmt* promoters, which are induced by the removal of thiamine (Maundrell, 1990; Maundrell, 1993). There are three different *nmt* promoters with varying strengths: *nmt1* , the strongest, *nmt41* , of intermediate strength, and *nmt81* , the weakest (Forsburg, 1993; Basi et al., 1993). A significant issue with *nmt* promoters is that they have a low dynamic range, inducing only about 10-fold above the repressed level of expression. There is considerable expression from these promoters even when repressed in the presence of thiamine, making them less suitable for specific experiments. Other promoters, such as ZEV have also been used to control the expression of genes in *S. pombe* . The advantage of the ZEV promoter is its titratable induction in the presence of different concentrations of beta-estradiol, with rapid induction (Ohira et al., 2017). However, ZEV also exhibits low dynamic range, comparable to the *nmt* promoters.

To address this issue, researchers have developed tetracycline-inducible promoters that exhibit tighter repression and, therefore, a greater dynamic range, approaching 100-fold (Faryar and Gatz, 1992; Erler et al., 2006; Zilio et al., 2012; Patterson et al., 2019). However, these tetracycline promoters do not achieve the high expression levels seen with the *nmt1* promoter. Another limitation of these earlier tetracycline promoters is that they require at least two different elements for the expression control: the promoter and the tetracycline repressor, which need to be transformed separately into yeast cells. To overcome these drawbacks, Lyu et al. modified the tetracycline promoter to create *PenotetS* , a system of five different promoters with different expression levels (Lyu et al., 2024).

Additionally, they developed a system in which both the promoter and tetracycline repressor are integrated on a single plasmid and are co-expressed. They used the fission yeast promoter *eno101* and built upon it by replacing the 19-bp sequence between the TATA box and TSS with the *tetO* sequence to create the *enotetS* promoter. They further developed four weaker derivatives of *PenotetS* by introducing GC-rich stem loops at the end of the 5' UTR. These promoters were named *PenotetSW1-SW4* (Lyu et al., 2024).

The expression level of the full strength *PenotetS* in the ‘on' state is similar to that of the *adh1* and *nmt1* promoters. In the 'off' state, the expression of *PenotetS* is 10-fold lower than that of *nmt1* , making it helpful in studying promoter shutoff phenotypes. Likewise, *PenotetSW2* 's expression resembles that of the *nmt41* promoter but with a much lower off-state expression. These promoters span a broad expression range: *SW2* 's level is tenfold lower than *PenotetS* , and *SW4* 's is one hundredfold lower. An additional benefit of *PenotetS* promoters over *nmt* promoters is faster induction. Whereas *nmt* promoters need about 16 hours of thiamine withdrawal to activate, *PenotetS* is activated quickly upon the addition of the inducer anhydrotetracycline (ahTet) and the expressed protein can reach significant levels within a hour (Lyu et al., 2024).

Given these advantages, these promoters help provide a range of expression levels to study various genes and their functions. However, the promoters developed by Lyu et al. are designed for studying genes expressed from ectopic loci. They are not suitable for studying endogenous genes at their genomic loci. To address this limitation, we developed plasmids that enable the replacement of endogenous promoters with *PenotetS* promoters.

We started by amplifying the *PenotetS* promoter cassettes, including the tetracycline repressor, tetracycline promoter, and mECitrine fluorescent tag. These sequences were integrated into three different drug-resistant cassettes (KanMX, HphMX, and NatMX). A total of 15 plasmids were constructed, comprising five different tetracycline promoters and three different drug-resistance cassettes. This collection allows the control of multiple genes with tetracycline promoters in a single yeast strain, providing flexibility through the use of different drug-resistance cassettes. Additionally, the constructs include an N-terminal mECitrine fluorescent tag. Using a single primer pair, one can replace a gene's endogenous promoter with any of the five *Penotet* promoters when inclusion of the mECitrine tag is desired. However, if the tag is not included, each *Penotet* promoter must be amplified using a distinct primer ( Figure 1A ).

To test the efficacy of these cassettes for promoter swapping, we selected the *wee1* gene, whose expression levels are reflected in cell length. Wee1 is an inhibitor of G2/M transition, and with increasing levels of Wee1 , cells grow longer in G2 (Russell and Nurse, 1987; Den Haese et al., 1995; Sveiczer et al., 2000; Keifenheim et al., 2017). This phenotype can be easily monitored by measuring the size, removing the need for fluorescent microscopy to assess protein expression. In the current study, we used *PenotetSW2* and *SW4* to replace the *wee1* promoter. When cells were grown in YES supplemented with 2.5µg/mL ahTet, a significant increase in cell length was observed compared to growth in the absence of ahTet ( Figure 1B ). Cell length at septation was measured under both conditions. In the case of the *PenotetSW2* :: *wee1* strain, cell lengths increased from 9.2±1.5 µm to 47 ±7.4 µm upon the addition of ahTet. Similarly, for the *PenotetSW4* :: *wee1* strain, cell length increased from 8±2.1 µm to 36±4.8 µm ( Figure 1C ). The results indicate variable levels of cell size increase, which correlate with the different promoter expression strengths. Under promoter shutoff conditions (in the absence of ahTet), both *PenotetSW2:: wee1* and *PenoteSW4:: wee1* showed tight repression, as evidenced by reduced Wee1 expression, which is reflected in significantly smaller cell lengths compared to the wild type. The length at septation for *PenotetSW4:: wee1* cells in the absence of ahTet closely resembled that of a *wee1* null mutant, where cell length at division is 7.6 µm (Russell and Nurse, 1987). These findings demonstrate that the dynamic range of tetracycline promoter cassettes is a powerful tool for studying promoter shutoff phenotypes of a desired gene.

## Methods

***S. pombe* Strains and Maintenance**

*S. pombe* strains were created using standard methods (Forsburg and Rhind, 2006) and grown in YES at 30°C. Anhydrotetracycline hydrochloride (J66688.MB, Thermo Fisher Scientific) was dissolved at a concentration of 5 mg/ml in DMSO, stored at -80°C and used at a concentration of 2.5 µg/ml.

**Plasmid Construction**

All plasmids were constructed by the Gibson Assembly method using the Gibson Assembly master mix (E2611S, NEB). The original tetracycline promoter plasmids pDB5318 ( *PenotetS* ,), pDB5319 ( *PenotetSW1* ), pDB5320 ( *PenotetSW2* ), pDB5321 ( *PenotetSW3* ), and pDB5322 ( *PenotetSW4* ) were procured from Addgene (https://addgene.com). PCR was performed with oligos SB239 (5'TGGCGAATGG GATGGCGGCGTTAGTATC3') and SB242 (5'AAAGATCTTACAGTTTAAACGAGCTCGAATTC3') to amplify plasmids comprising drug-resistant cassette KanMX (pFA6a-kanMX6), HphMX (pCR2.1-hph) or NatMX (pCR2.1-nat). To amplify tetracycline promoters, including tetracycline repressor and mECitrine sequences, PCR was done with oligos SB240 (5' CGCCGCCATCCCATTCGCCATTCAGGCTG3') and SB243 (5'GTTTAAACTGTAAGATCTTTTGTATAGTTCATCCATGC3'). A single oligo pair was used to amplify all tetracycline promoters (including mECitrine and tet repressor), which were cloned downstream of the KanMX, HphMX, and NatMX cassettes. These plasmids were named as listed in Table 1. All plasmids generated in this study were sequenced by Plasmidsaurus ( https://plasmidsaurus.com/ ). Plasmids created in this study will be available from Addgene (https://addgene.com) and NRBP (https://yeast.nig.ac.jp/yeast/fy/StrainAllItemsList.jsf).

**Strain Construction**

To construct *wee1* promoter replacement strains, yFS110 was transformed with the following PCR products: for the *PenotetSW2* promoter swap, PCR was performed on pFS558 with oligos MO600 (5'GCATTCCAATTCAATTTAATTAAATCAAAAATTTCA TATCTATTTTTTTGTT AAATTGCCACATTTTCCATACAGAAAA *cgacatggaggcccagaa* 3') and MO603 (5'GCACGATTTAGATTCATGGAGCGTTGG GACCGCCGTAAGCCATAAG ATCTATGACTGCTGGTATTAGAAGAAGAG *ctcatgctagcggcccgag cggccc* 3'), and for *PenotetSW4* , PCR was performed on pFS560 with oligos MO600 and MO602 (5'GCACGATTTAGATTCATGGAGCGTTGGGACCGCCGTAAGCCATAAGATCTATGACT GCTGGTATTAGAAGAAGAGCTCA *tcatgctagccgggcccgagc* 3'). Oligo regions that bind to the plasmid are shown in lowercase italics, while regions that target the *wee1* genomic sequence are shown in uppercase. PCR products were transformed using electroporation (Torres-Garcia et al., 2020). Transformants were selected on YES Hygromycin (50mg/ml). Strains created in this study will be available from NRBP ( https://yeast.nig.ac.jp/yeast/fy/StrainAllItemsList.jsf ).

## Reagents

**Table:** 

**Strain Name**	**Genotype**	**Reference**
yFS110	h- leu1-32 ura4-D18 ade6-216 his7-366	lab stock
yFS1161	h- leu1-32 ura4-D18 ade6-216 his7-366 PenotetSW2: Wee1 ::HPHMX	this paper
yFS1162	h- leu1-32 ura4-D18 ade6-216 his7-366 PenotetSW4: Wee1 ::HPHMX	this paper
**Plasmid Name**	**Description**	**Reference**
pFA6a-kanMX6	KanMX drug cassette	(Bähler et al., 1998)
pCR2.1-hph	HphMX drug cassette	(Sato et al., 2005)
pCR2.1-nat	NatMX drug cassette	(Sato et al., 2005)
pDB5318	*PenotetS* promoter	(Lyu et al., 2024)
pDB5319	*PenotetSW1* promoter	(Lyu et al., 2024)
pDB5320	*PenotetSW2* promoter	(Lyu et al., 2024)
pDB5321	*PenotetSW3* promoter	(Lyu et al., 2024)
pDB5322	*PenotetSW4* promoter	(Lyu et al., 2024)
pFS551	KanMX *PenotetS* promoter cassette	this paper
pFS552	KanMX *PenotetSW1* promoter cassette	this paper
pFS553	KanMX *PenotetSW2* promoter cassette	this paper
pFS554	KanMX *PenotetSW3* promoter cassette	this paper
pFS555	KanMX *PenotetSW4* promoter cassette	this paper
pFS556	HphMX *PenotetS* promoter cassette	this paper
pFS557	HphMX *PenotetSW1* promoter cassette	this paper
pFS558	HphMX *PenotetSW2* promoter cassette	this paper
pFS559	HphMX *PenotetSW3* promoter cassette	this paper
pFS560	HphMX *PenotetSW4* promoter cassette	this paper
pFS561	NatMX *PenotetS* promoter cassette	this paper
pFS562	NatMX *PenotetSW1* promoter cassette	this paper
pFS563	NatMX *PenotetSW2* promoter cassette	this paper
pFS564	NatMX *PenotetSW3* promoter cassette	this paper
pFS565	NatMX *PenotetSW4* promoter cassette	this paper
